# Possible Avoidant Personality Disorder Magnifies the Association Between Bullying Victimization and Depressive Symptoms Among Chinese University Freshmen

**DOI:** 10.3389/fpsyt.2022.822185

**Published:** 2022-02-17

**Authors:** Yan-Min Xu, Shan-Shan Pu, Yi Li, Bao-Liang Zhong

**Affiliations:** ^1^Wuhan Mental Health Center, School of Mental Health and Psychological Sciences, Anhui Medical University, Wuhan, China; ^2^Department of Psychiatry, Wuhan Mental Health Center, Wuhan, China

**Keywords:** bullying victimization, depressive symptoms, avoidant personality, freshmen, China

## Abstract

**Background:**

Bullying victimization has been associated with depressive symptoms in Chinese university students. This study examined the moderating effect of possible avoidant personality disorder (APD) on association between bullying victimization and depressive symptoms in university freshmen.

**Methods:**

A total of 1,453 freshmen were recruited from a comprehensive university in Wuhan, China, and administered a self-report questionnaire. The APD subscale of Personality Diagnostic Questionnaire–Version 4 and Beck Depression Inventory were used to measure the presence of possible APD and depressive symptoms, respectively. The moderating effect of possible APD was examined by testing the statistical significance of the interaction between victimization and possible APD in multiple logistic regression analysis.

**Results:**

The prevalence of depressive symptoms was 24.8%. In multiple logistic regression analysis, the interaction between bullying victimization and possible APD was significantly associated with depressive symptoms (OR: 1.80, *P* = 0.029). Subsequent subgroup analyses according to the status of possible APD showed that, the victimization-depression association was stronger among freshmen with possible APD (OR: 3.23, *P* < 0.001) than those without possible APD (OR: 1.82, *P* = 0.001).

**Conclusion:**

In Chinese university freshmen, bullying victimization is significantly associated with depressive symptoms, and possible APD magnifies the victimization-depression association. Bullied freshmen, particularly freshmen with possible APD, could be considered as the target group of campus-based depression intervention programs.

## Introduction

The transitioning from junior middle schools to universities is challenging for most university freshmen because of difficulties in adjusting to new surroundings, forming new self-identities, forging friendships, gaining independence, and overcoming academic pressure (1, 2). Accordingly, studies have shown the high risk of mental health problems in first-year university students, in particular depression; for example, as high as 35.4–41.4% of the Chinese university freshmen suffer from depressive symptoms (3, 4).

The etiology of depression is complex and multifactorial, and, in the population of university students, commonly reported factors associated with depressive symptoms were female sex, rural origin, low family income, inadequate social support, loneliness, negative life events, academic stress, and certain personality features such as high neuroticism (3–6). Despite extensive research on the epidemiology of depressive symptoms in university students, few studies have examined mechanisms linking these factors and depressive symptoms.

Bullying victimization refers to exposure to aggressive behavior repeatedly and over time from one or more perpetrators who have greater physical or social power than their victims (7). Convincing evidence has shown the causal association between being bullied and mental health problems in children and adolescents including anxiety, depression, non-suicidal self-injury, and suicidal behaviors (8). Although traditional bullying such as verbal and physical bullying is primarily recognized as a public health problem in primary and middle school students, bullying victimization is also prevalent among university students (9). For example, as high as 88.2% of the Chinese university freshmen experienced verbal bullying in the past 12 months and Chinese university students who are bullied are two times more likely than those who are not bullied to have depressive symptoms (9, 10).

The vulnerability–stress model of depression argues that personality interacts with stressors to influence the development of depressive symptoms (11–13). Several subtypes of personality psychopathology have been associated with the increased risk of major depression such as depressive, borderline, and avoidant personality disorders (14–17). In the literature, the mechanisms underlying associations between depressive and borderline personality disorders and depressive disorders have been extensively examined, but the way in which avoidant personality disorder (APD) increases the risk of depression remains poorly understood (18–20).

Persons with APD avoid intimate and social contact with others, are sensitive to negative criticism and rejection, and have a high level of fear of being ridiculed by others (20). Findings from a comparative study have shown that compared to university students without APD, those with APD have significantly lower levels of self-esteem and, therefore, are more likely to experience negative affect (21).

In Chinese universities, although only 0.54–0.66% of the students meet the DSM-IV diagnostic criteria for APD (22, 23), 30.4–33.4% of the students have possible APD, as defined by the APD subscale of Personality Diagnostic Questionnaire–Version 4 (PDQ-4), a widely used screener of personality disorders (24–26). Despite not a clinical diagnosis of APD, possible APD has been found to be a stable personality trait in Chinese university students and the severity of depressive symptoms is significantly associated with the APD subscale score of PDQ-4 in this population (26, 27). Because of the vulnerability of persons with APD, we speculated that Chinese university students with possible APD would experience more depressive symptoms if they were bullied. Since understanding the contextual roles of APD and other personality disorders on the relationship between bullying victimization and depressive symptoms would facilitate the development of campus-based depression prevention programs in Chinese universities, this study examined the moderating role of possible APD on the bullying-depression association in Chinese university freshmen, that is, whether the negative effect of bullying victimization on depressive symptoms differed between students with and without possible APD.

## Materials and Methods

### Participants

This study was part of a large-scale survey, which investigated mental health, quality of life, and personality among fresh students of a comprehensive university in Wuhan, China, between November and December 2019. Eligible respondents were first-year undergraduates who were of Chinese nationality and voluntary to join the study. We randomly selected seven schools from a total of 19 schools of the university and invited all the freshmen to participate in the survey.

The Ethics Committee of Wuhan Mental Health Center approved the study protocol. All respondents signed the informed consent before the formal survey.

### Procedures and Instruments

We used a standardized self-administered questionnaire to gather data on sociodemographics, lifestyle, experience of being bullied, and depressive symptoms.

Sociodemographic factors included sex, age, ethnic group, place of origin (urban vs. rural), living arrangement (with roommates vs. with family members), self-rated family financial status (good, moderate, poor), the only-child status, and marital status of parents (married vs. others).

Lifestyle factors included current drinking and current smoking. According to the official manual of the Chinese Adolescent Health-Related/Risk Behavior Surveillance, current drinkers and smokers were defined as individuals who consumed any alcoholic beverage and smoked at least once in the past month, respectively (28).

Questions for assessing bullying victimization were adapted from previous school bullying studies in China (29–31), which asked respondents about the presence of any one of the seven subtypes of traditional bullying victimization at universities during any time of the past 12 months: “been teased in a mean or hurtful way,” “been asked for money or other possessions in a mean or hurtful way,” “been left out of a group or ignored on purpose in a mean or hurtful way”, “been threatened or intimidated in a mean or hurtful way,” “been hit, kicked, pushed, squeezed, or locked in a mean or hurtful way,” “had sexual jokes or gestures made to you in a mean or hurtful way,” and “had your things stolen or damaged in a mean or hurtful way”.

We used the APD subscale of the validated Chinese PDQ-4 to identify the possible presence of APD among respondents (32). The subscale has seven self-report questions and responses are in a true/false (1/0) format. According to previous studies (24, 33), a total score of four or higher was used to denote possible APD in Chinese university students.

Depressive symptoms were measured with the validated Chinese 21-item Beck Depression Inventory, the Second Edition (BDI-II), which is one of the most widely used scales for assessing the severity of depressive symptoms in both clinical and non-clinical populations in China, including university students (34–36). All BDI-II items use a 0–3 scale to rate the magnitude to which a persons has experienced each depressive symptom during the past 2 weeks. The total score ranges between zero and 63 with a cut-off score of 14 or more denoting the presence of depressive symptoms.

### Statistical Analysis

Rates of depressive symptoms were calculated and comparisons between/across subgroups according to sociodemographics were made using the univariate logistic regression analysis. To examine the independent victimization-depression association, a multiple logistic regression analysis was performed, which entered bullying victimization as the main predictor; possible APD, sociodemographic factors, and lifestyle factors as the covariates; and depressive symptoms as the outcome variable (“main effect model”). To test whether the victimization-depression association differed between students with and without possible APD, an interaction term, the production of victimization and possible APD, was included as an additional independent variable in the main effect model (“interactive effect model”). A statistically significant interaction term suggested the presence of the moderating effect of possible APD on the victimization-depression association. Finally, we repeated the above multiple logistic regression analyses within samples with and without possible APD to estimate the adjusted victimization-depression associations in the contexts of the presence and absence of possible APD, respectively. We quantified the factor-outcome association by using odds ratio (OR) and its 95% confidence interval (CI). All statistical analyses were performed by using SPSS 25.0, assuming a two-sided test at the 0.05 level of statistical significance.

## Results

Altogether, 1,557 freshmen were invited and 1,453 completed the survey questionnaire (response rate: 93.3%). Among the completers, 868 (59.7%) were boys and the mean age was 20.3 years (standard deviation: 0.9, range: 16–26). The prevalence rates of bullying victimization, possible APD, and depressive symptoms were 39.2, 31.7, and 24.8%, respectively. Other detailed sociodemographic and lifestyle characteristics of the study sample are displayed in Table 1.

**Table 1:** Relationships across bullying victimization, possible avoidant personality disorder (APD), and depressive symptoms among Chinese university freshmen.

**Variables**		***n***	**Number of depressed** **students (%)**	**Univariate logistic regression analysis**		**Multiple logistic regression analysis**		**Multiple logistic regression analysis with the interaction term**		**Multiple logistic regression analysis in sample without possible APD**		**Multiple logistic regression analysis in sample with possible APD**	
				**Odds ratio (95% CI)**	***P***	**Odds ratio (95% CI)**	***P***	**Odds ratio (95% CI)**	***P***	**Odds ratio (95% CI)**	***P***	**Odds ratio (95% CI)**	***P***
Bullying victimization × Possible APD								1.80 (1.06, 3.04)	0.029				
Sex	Male	868	200 (23.0)	1		1		1		1		1	
	Female	585	161 (27.5)	1.27 (0.99, 1.61)	0.053	1.54 (1.17, 2.04)	0.002	1.55 (1.17, 2.06)	0.002	1.55 (1.17, 2.06)	0.039	1.56 (1.02, 2.39)	0.040
Age-group (years)	≤ 20	861	216 (25.1)	1		1		1		1		1	
	>20	592	145 (24.5)	0.97 (0.76, 1.24)	0.797	0.98 (0.75, 1.28)	0.877	0.98 (0.75, 1.28)	0.871	0.83 (0.58, 1.20)	0.316	1.18 (0.79, 1.77)	0.417
Ethnic group	Han	1,339	330 (24.6)	1		1		1		1		1	
	Minority	114	31 (27.2)	1.14 (0.74, 1.76)	0.546	1.25 (0.78, 2.00)	0.346	1.28 (0.80, 2.04)	0.306	1.64 (0.93, 2.92)	0.089	0.83 (0.38, 1.80)	0.636
Place of origin	Urban	1,050	246 (23.4)	1		1				1		1	
	Rural	403	115 (28.5)	1.31 (1.01, 1.69)	0.044	1.22 (0.88, 1.68)	0.238	1.22 (0.88, 1.70)	0.229	1.22 (0.79, 1.90)	0.375	1.29 (0.79, 2.11)	0.313
Living arrangement	With roommates	1,418	349 (24.6)	1		1		1		1		1	
	With family members	35	12 (34.3)	1.60 (0.79, 3.25)	0.195	1.68 (0.76, 3.71)	0.201	1.67 (0.76, 3.68)	0.202	2.42 (0.93, 6.29)	0.070	0.88 (0.25, 3.11)	0.843
Self-rated family financial status	Good	299	75 (24.1)	1		1		1		1		1	
	Moderate	1,027	242 (23.6)	0.92 (0.68, 1.24)	0.588	1.12 (0.80, 1.55)	0.518	1.11 (0.80, 1.55)	0.422	1.12 (0.70, 1.78)	0.642	1.11 (0.69, 1.80)	0.671
	Poor	127	44 (34.6)	1.58 (1.01, 2.48)	0.045	1.64 (0.94, 2.85)	0.083	1.60 (0.91, 2.80)	0.101	1.31 (0.59, 2.92)	0.513	1.98 (0.87, 4.50)	0.104
The only child	No	373	92 (24.7)	1		1		1		1		1	
	Yes	1,080	269 (24.9)	1.01 (0.77, 1.33)	0.926	1.32 (0.93, 1.87)	0.119	1.35 (0.95, 1.91)	0.094	1.30 (0.89, 2.06)	0.275	1.55 (0.90, 2.66)	0.113
Marital status of parents	Married	1,363	326 (23.9)	1		1		1				1	
	Others*	90	35 (38.9)	2.02 (1.30, 3.15)	0.002	1.83 (1.12, 2.99)	0.016	1.87 (1.15, 3.06)	0.012	1.97 (1.04, 3.74)	0.038	1.87 (0.87, 4.02)	0.108
Current drinking	No	884	210 (23.8)	1		1		1				1	
	Yes	569	151 (26.5)	1.16 (0.91, 1.48)	0.231	1.28 (0.97, 1.68)	0.085	1.28 (0.97, 1.69)	0.08	1.36 (0.94, 1.98)	0.105	1.18 (0.78, 1.80)	0.436
Current smoking	No	1,379	339 (24.6)	1		1		1				1	
	Yes	73	22 (30.1)	1.33 (0.79, 2.22)	0.286	1.19 (0.67, 2.11)	0.550	1.21 (0.68, 2.16)	0.512	1.44 (0.69, 3.01)	0.327	0.95 (0.39, 2.29)	0.901
Bullying victimization	No	883	159 (18.0)	1		1		1				1	
	Yes	570	202 (35.4)	2.50 (1.96, 3.19)	<0.001	2.39 (1.84, 3.10)	<0.001	1.84 (1.29, 2.61)	0.001	1.82 (1.28, 2.59)	0.001	3.23 (2.17, 4.82)	<0.001
Possible APD	No	993	155 (15.6)	1		1							
	Yes	460	206 (44.8)	4.39 (3.41, 5.64)	<0.001	4.20 (3.24, 5.44)	<0.001	3.17 (2.21, 4.54)	<0.001				

* *Others included never-married, remarried, cohabiting, separated, divorced, and widowed*.

In both the univariate analysis and the main effect model, both bullying victimization and possible APD were significantly associated with depressive symptoms (OR: 2.50 and 4.39, *P* < 0.001; OR: 2.39 and 4.20, *P* < 0.001). In the interactive effect model, the interaction between bullying victimization and possible APD was significantly associated with depressive symptoms (OR: 1.80, *P* = 0.029). In the subsequent subgroup analyses according to the status of possible APD, the victimization-depression association was stronger among freshmen with possible APD (OR: 3.23, *P* < 0.001) than those without possible APD (OR: 1.82, *P* = 0.001) (Table 1).

## Discussion

To the best of our knowledge, this is the first study in China that examined both the victimization-depression association and the moderating effect of possible APD on the victimization-depression association among university freshmen. Compared to the 8.0% 12-month prevalence of bullying victimization in college students in Changsha, China (9), we found a much higher 12-month prevalence of bullying victimization in university freshmen (39.2%). However, due to the heterogeneity in definitions and timeframes of depressive symptoms, prevalence rate of depressive symptoms in our study are lower than those reported in prior studies (24.8% vs. 35.4–41.4%) (3, 4). Nevertheless, the figure, nearly one-fourth of the freshmen were depressed, still indicates that depressive symptoms are a substantial mental health problem in Chinese university freshmen and there is an urgent need for developing intervention programs to prevent or reduce depression in this population.

In the sample of Chinese university freshmen, we observed significant and independent associations of depressive symptoms with bullying victimization and possible APD, which are consistent with findings from studies with middle school students and other populations (8, 26). The significant victimization-APD interactive effect on depression and greater victimization-depression association in freshmen with than those without possible APD suggest that possible APD magnifies the negative effect of bullying victimization on emotional health in university freshmen. Since the interactive effect was independent from sociodemographic and lifestyle factors, we speculated that some core features of APD may explain the moderating role of possible APD on victimization-depression association. First, social support can buffer the negative effect of a stressful event on the mental health of individuals (37). Because persons with APD avoid social contact with others and are more likely to have insufficient social support to cope with stressful situations, freshmen with possible APD would be more likely to be depressed if they were bullied. Second, persons with APD are overly concerned with “looking foolish” and tend to adopt avoidance coping strategies to deal with stressful situations, so bullied students are more likely to be stressed and depressed if they had possible APD.

This stud has two limitations. First, our assessment of bullying victimization did not include cyberbullying victimization, which is also prevalent and associated with depression in university students (38). So the current study can not examine the moderating effect of possible APD on association between cyberbullying victimization and depression. Second, the sample was selected from a comprehensive university in Wuhan, China, and fresh students from universities of other cities and other categories (i.e., medical) were not included, so there might be selection bias in the study sample. Third, some psychosocial factors associated with depressive symptoms such as social support and possible dependent personality were not measured and included in the adjustment analysis. Further studies are needed to exclude the confounding effects of these unmeasured psychosocial factors.

In summary, in Chinese university freshmen, bullying victimization is significantly associated with depressive symptoms, and possible APD magnifies the victimization-depression association. Bullied freshmen, particularly freshmen with possible APD, could be considered as the target group of campus-based depression prevention programs. In addition, campus-based programs designed to prevent depression in freshmen may include bullying intervention program, psychosocial support, social skills training, and stress management training.

## Data Availability Statement

The raw data supporting the conclusions of this article will be made available by the authors, without undue reservation.

## Ethics Statement

The studies involving human participants were reviewed and approved by Ethics Committee of Wuhan Mental Health Center. The patients/participants provided their written informed consent to participate in this study.

## Author Contributions

Y-MX: acquisition and analysis of data for the study, drafting the paper, and interpretation of data for the study. Y-MX and S-SP: design and acquisition of data for the study. YL and B-LZ: drafting the paper, revising the paper for important intellectual content, and interpretation of data for the study. All authors contributed to the article and approved the submitted version.

## Funding

This work was supported by National Natural Science Foundation of China (Grant Number: 71774060). The funding source listed had no role in study design; in the collection, analysis, and interpretation of data; in the writing of the report; and in the decision to submit the paper for publication.

## Conflict of Interest

The authors declare that the research was conducted in the absence of any commercial or financial relationships that could be construed as a potential conflict of interest.

## Publisher's Note

All claims expressed in this article are solely those of the authors and do not necessarily represent those of their affiliated organizations, or those of the publisher, the editors and the reviewers. Any product that may be evaluated in this article, or claim that may be made by its manufacturer, is not guaranteed or endorsed by the publisher.
